# Effects of Regulating Hippo and Wnt on the Development and Fate Differentiation of Bovine Embryo

**DOI:** 10.3390/ijms25073912

**Published:** 2024-03-31

**Authors:** Peipei Zhang, Hang Zhang, Chongyang Li, Baigao Yang, Xiaoyi Feng, Jianhua Cao, Weihua Du, Muhammad Shahzad, Adnan Khan, Shao-Chen Sun, Xueming Zhao

**Affiliations:** 1 Institute of Animal Sciences (IAS), Chinese Academy of Agricultural Sciences (CAAS), No. 2 Yuanmingyuan Western Road, Haidian District, Beijing 100193, China; 82101209707@caas.cn (P.Z.); 82101215397@caas.cn (H.Z.); 15652652378@163.com (C.L.); 82101211205@caas.cn (B.Y.); 17806257712@163.com (X.F.); jhcao0323@126.com (J.C.); duweihua@caas.cn (W.D.); drmshahzadvet@gmail.com (M.S.); 2 College of Animal Science and Technology, Nanjing Agricultural University, Nanjing 210095, China; sunsc@njau.edu.cn; 3 Genome Analysis Laboratory of the Ministry of Agriculture, Agriculture Genomics Institute at Shenzhen, Chinese Academy of Agricultural Sciences, Shenzhen 518120, China; dr.adnan93@gmail.com

**Keywords:** LPA, DKK1, embryo, single-cell RNA sequencing

## Abstract

The improvement of in vitro embryo development is a gateway to enhance the output of assisted reproductive technologies. The Wnt and Hippo signaling pathways are crucial for the early development of bovine embryos. This study investigated the development of bovine embryos under the influence of a Hippo signaling agonist (LPA) and a Wnt signaling inhibitor (DKK1). In this current study, embryos produced in vitro were cultured in media supplemented with LPA and DKK1. We comprehensively analyzed the impact of LPA and DKK1 on various developmental parameters of the bovine embryo, such as blastocyst formation, differential cell counts, YAP fluorescence intensity and apoptosis rate. Furthermore, single-cell RNA sequencing (scRNA-seq) was employed to elucidate the in vitro embryonic development. Our results revealed that LPA and DKK1 improved the blastocyst developmental potential, total cells, trophectoderm (TE) cells and YAP fluorescence intensity and decreased the apoptosis rate of bovine embryos. A total of 1203 genes exhibited differential expression between the control and LPA/DKK1-treated (LD) groups, with 577 genes upregulated and 626 genes downregulated. KEGG pathway analysis revealed significant enrichment of differentially expressed genes (DEGs) associated with TGF-beta signaling, Wnt signaling, apoptosis, Hippo signaling and other critical developmental pathways. Our study shows the role of LPA and DKK1 in embryonic differentiation and embryo establishment of pregnancy. These findings should be helpful for further unraveling the precise contributions of the Hippo and Wnt pathways in bovine trophoblast formation, thus advancing our comprehension of early bovine embryo development.

## 1. Introduction

The development of mammalian embryos progresses from the zygotic stage to the blastocyst and includes processes such as embryo genome activation (EGA), the determination of cell lineages and the differentiation of cell fates [1]. The embryonic genome is activated at a certain point in embryonic development [2]. This development proceeds independently of the maternal genome, leading to the formation of three distinct cell lineages: the trophectoderm (TE), primitive endoderm (PE) and epiblast (EPI) [3]. The initial specific lineage differentiation occurs when the outer cells form the TE [4]. Subsequently, the inner cell mass (ICM) differentiates into PE and EPI cells, marking the second lineage-specific differentiation [5]. Finally, the TE gives rise to extraembryonic tissues, while the PE and EPI cells develop into the extraembryonic yolk sac and the actual embryo, respectively [6]. 

Wnt signaling is pivotal in orchestrating the developmental dynamics of the preimplantation embryo [7]. It is implicated in a myriad of developmental processes such as cellular differentiation [8,9] and proliferation [10], as well as lineage specification and the sustenance of pluripotency [11]. Furthermore, Wnt signaling is integral to axial elongation [12], the establishment of cell polarity and motility, and the regulation of epithelial–mesenchymal transition [13]. The endometrial secretory protein Dickkopf-1 (DKK1), a key mediator in maternal–embryonic crosstalk, inhibits the Wnt pathway by disrupting the formation of the Wnt ligand/Frizzled receptor/LRP5 or LRP6 complex, thereby facilitating embryonic differentiation [11,14,15]. 

The Hippo signaling pathway is highly conserved in mammals and is involved in follicle growth and follicular activation [16,17,18], and it has been shown to play a key role in embryonic development [19]. Recently, studies have elucidated the critical involvement of the Hippo-YAP pathway in the differentiation of the TE and ICM during preimplantation embryonic development [20]. The transcriptional coactivator YAP is implicated in embryonic development and organogenesis, which can also enhance cell proliferation and prevent apoptosis [21]. Lysophosphatidic acid (LPA) is a biologically active phospholipid that regulates a wide range of cellular effects through the activation of specific G protein-coupled receptors [22]. LPA-mediated signaling has been shown to play a key role in mouse embryo spacing and implantation time [23]. LPA is present in all mammalian cells and tissues, inducing cell proliferation, survival and migration [24,25,26].

The implementation of single-cell RNA sequencing (scRNA-seq) technology now facilitates the detailed characterization of single-cell transcriptomes across various developmental stages [27,28]. With the utilization of scRNA-seq technology, recent studies have elucidated the molecular features of gastrulation and organogenesis during the early stages of mouse development, as well as the cellular mechanisms in heart development [29,30]. Meanwhile, the effects of vitrification on sheep embryos have been analyzed at the single-embryo transcriptome level by scRNA-seq [31]. However, to our best knowledge, a comprehensive understanding of the signaling pathways and regulatory mechanism involved in early bovine embryo development at single-cell resolution is still lacking.

In this study, we analyzed the regulation of Hippo and Wnt signaling pathways by LPA and DKK1 supplemented in in vitro culture (IVC) medium, and how this affects the development of bovine embryos. The findings of this study will contribute to establishing an efficient approach to improve the developmental capacity of bovine embryos.

## 2. Results

### 2.1. Effect of LPA and DKK1 on the Development of Bovine Embryos 

To examine how the Hippo and Wnt pathways are involved in embryo development, bovine oocytes were fertilized in vitro and then cultured to the blastocyst stage in the presence of LPA and DKK1. The cleavage rate and the blastocyst development rate of the LPA + DKK1 group (89.67 ± 4.53%, 47.11 ± 4.23%) were significantly higher than those of the LPA group (83.57 ± 3.60%, 40.04 ± 2.54%), the DKK1 group (81.97 ± 3.38%, 38.91 ± 3.10%) and the control group (74.18 ± 3.44%, 30.02 ± 3.30%; *p* < 0.05), as presented in Table 1.

### 2.2. Effect of LPA and DKK1 on the YAP Fluorescence Intensity of Bovine Embryos 

**Figure 1:**
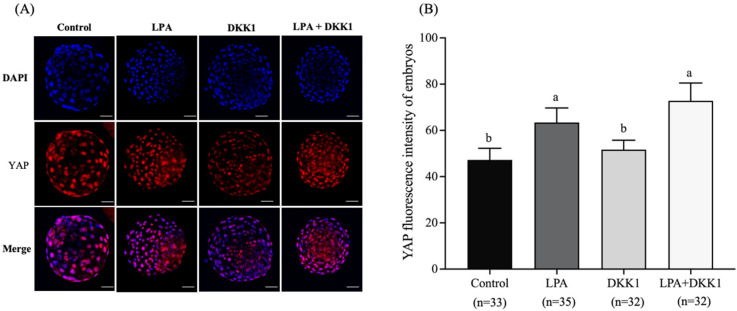
Effect of LPA and DKK1 on the YAP fluorescence intensity of bovine embryos. (**A**) YAP immunofluorescence in the bovine embryos; (**B**) Effect of LPA and DKK1 on the YAP fluorescence intensity of bovine embryos. Scale bar = 50 μm. a, b Values with different superscripts indicate significant difference between groups (*p* < 0.05).

As shown in Figure 1, the YAP fluorescence intensity of the LPA group (63.41 ± 5.61) and the LPA + DKK1 group (72.81 ± 6.84) were significantly higher compared to the control group (47.21 ± 4.53) and the DKK1 group (51.66 ± 3.65, *p* < 0.05).

### 2.3. LPA and DKK1 Enhanced the Number of TE Cells of Bovine Embryos

**Figure 2:**
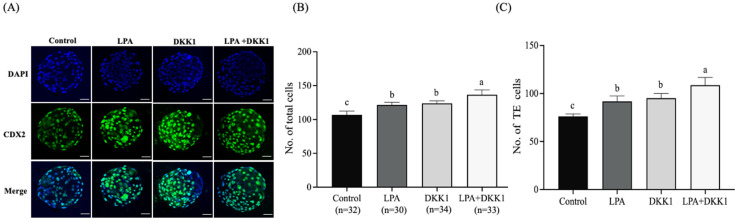
LPA and DKK1 increase number of TE cells of bovine embryos. (**A**) Fluorogram of CDX2-stained bovine embryos; (**B**) total cell numbers of bovine embryos; (**C**) TE cell numbers of bovine embryos. Scale bar = 50 μm. a, b, c Values with different superscripts indicate significant difference between groups (*p* < 0.05).

We evaluated the changes in the development and differentiation of embryonic TE cells with LPA and DKK1 treatments. As shown in Figure 2, the results showed that the total cell numbers and the TE cell numbers of the LPA group (121.63 ± 3.38, 91.82 ± 5.00), the DKK1 group (123.85 ± 3.40, 95.22 ± 4.28) and the LPA + DKK1 group (136.67 ± 6.52, 108.70 ± 7.27) were significantly higher compared to the control group (107.00 ± 4.82, 76.23 ± 2.31; *p* < 0.05).

### 2.4. LPA and DKK1 Suppressed the Apoptosis of Bovine Embryos 

**Figure 3:**
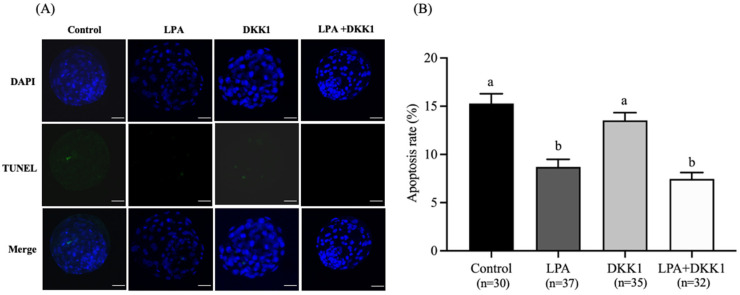
Effect of LPA and DKK1 on the apoptosis of bovine embryos. (**A**) Fluorogram of TUNEL-stained embryos; (**B**) Effect of LPA and DKK1 on the apoptosis of bovine embryos. Scale bar = 50 μm. a, b Values with different superscripts indicate significant difference between groups (*p* < 0.05).

As shown in Figure 3, the apoptosis rates of blastocysts in the LPA group (8.63 ± 0.68%) and LPA + DKK1 group (7.45 ± 0.59%) were significantly lower compared to the control group (15.28 ± 0.91%) and the DKK1 group (13.53 ± 0.71%, *p* < 0.05).

### 2.5. Transcriptomic Analysis of LPA- and DKK1-Treated Bovine Embryos

Using scRNA-seq for single embryos, the results of quality control are shown in Table 2; the expression level of each sample met the quality control requirements. We obtained approximately 52 million sequencing clean reads from duplicate samples of bovine blastocyst. The clean reads numbers of the control groups ranged from 44,891,384 to 62,536,622 and the clean reads rates were higher than 98.09%; the clean reads numbers of the LD groups varied from 40,869,626 to 54,470,954 and the clean reads rates were higher than 97.88%. The mapped ratios of the control group were higher than 97.51%, and the mapped ratios of the LD group were higher than 97.43%. 

### 2.6. Screening of Differentially Expressed Genes (DEGs) in LPA- and DKK1-Treated Bovine Embryos

**Figure 4:**
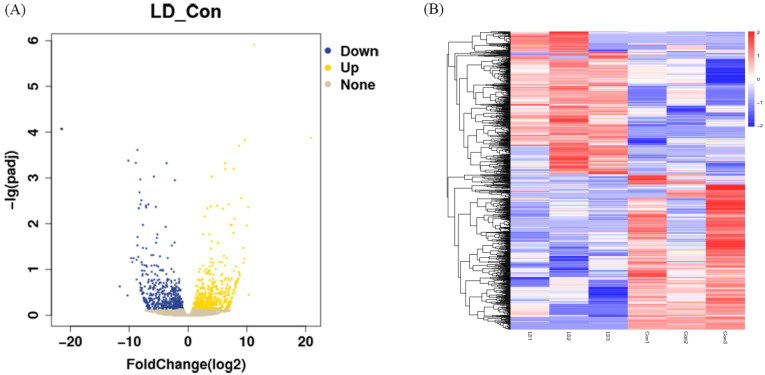
Screening of differential expression of RNA. (**A**) Volcano plot of DEGs in LD and control groups. (**B**) Clustering analysis of differentially expressed genes. Red indicates high expression and blue indicates low expression.

Differences in gene expression levels between the LD and control groups were depicted using volcano plots (Figure 4A). A total of 1203 DEGs were found between the LD and control groups, including 577 upregulated genes and 626 downregulated genes. Some of those DEGs are listed in Table 3, including apoptosis genes (*BAD*, *FAS*, *BEX2*), oxidative stress genes (*GPX3*, *SLC25A27*, *MGST1*), embryo development genes (*ASTL*, *ABCB4*, *EED*, *TGFBR2*, *IGFBP7*), endometrial-related genes (*MX2*, *c15H11orf34*, *MUC1*, *COL5A3*) and embryonic differentiation genes (*FOS*, *GCM1*, *OVOL1*, *KRT7*). The full list is provided in Supplementary Table S1. Cluster analysis of the DEGs was then performed according to the expression, and results showed that samples in the same group were well clustered together (Figure 4B). 

### 2.7. GO Enrichment Analysis of the DEGs

**Figure 5:**
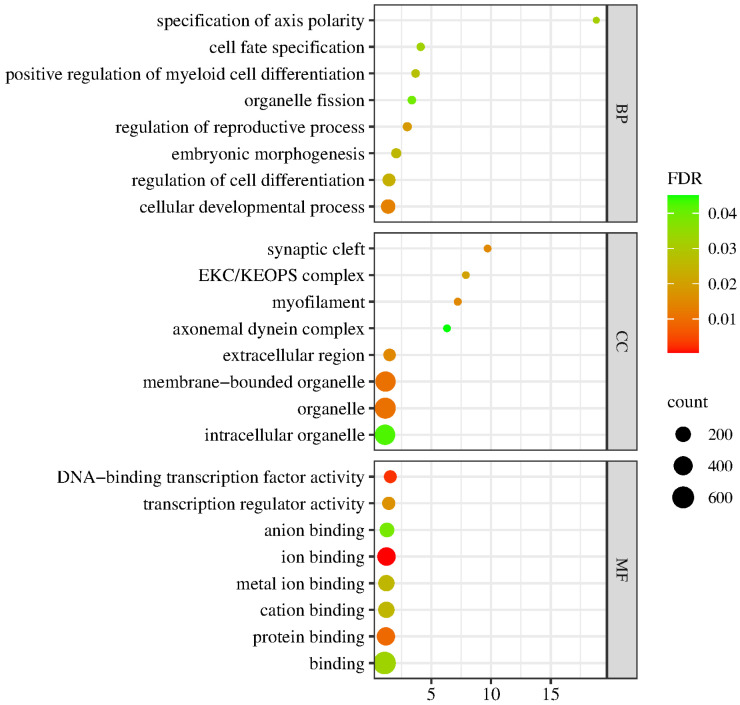
GO enrichment analysis of the DEGs. The spot color represents the FDR, and the spot size represents the enriched gene number.

Differential expression analysis, functional annotation and functional enrichment analysis of DEGs were performed according to the expression level of the genes in the LD and control groups. As shown in Figure 5, the GO terms for genes associated with the DEGs between the LD and control groups were mainly involved in biological process (BP), cell component (CC) or molecular function (MF). The BP group mainly consisted of cell fate specification, regulation of cell differentiation and cellular development process; CC mainly consisted of synaptic cleft, myofilament, extracellular region and organelle; and MF mainly consisted of DNA-binding transcription factor activity, transcription regulator activity, ion binding and protein binding.

### 2.8. Significantly Regulated Biological Signaling Pathways in Response to LPA and DKK1 Treatment in Bovine Embryos

**Figure 6:**
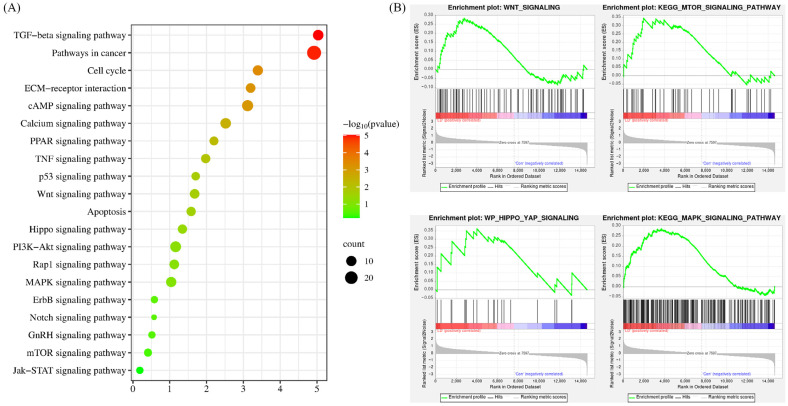
KEGG pathway analysis of the DEGs. (**A**) Results from KEGG analysis; the color of each dot indicates the *p* value of the two sets of DEGs, and the size of the dot indicates the number of DEGs in each KEGG pathway. (**B**) The results from GSEA analysis.

To enhance our comprehension of the signaling pathways regulated by LPA and DKK1 treatment, the identified DEGs were subjected to pathway analysis utilizing the KEGG. As shown in Figure 6A, the DEGs’ associated genes between the LD and control groups were mainly enriched with TGF-beta signaling pathway, ECM-receptor interaction, Wnt signaling pathway, Apoptosis, Hippo signaling pathway, MAPK signaling pathway and Notch signaling pathway. The overall changes in the gene set of the LD group analyzed by GSEA are shown in Figure 6B.

## 3. Discussion

Culture environments typically control embryo cleavage, compaction, survival and development [32]. To elucidate the impact of IVC conditions on embryonic quality, the culture media were supplemented with the Hippo pathway agonist (LPA) and the Wnt pathway inhibitor (DKK1). Many research studies have endeavored to optimize culture systems to enhance blastocyst quality. Earlier studies have found that LPA treatment increases the abundance of transcripts of Connexin 43, GJC1 and CDH1, which would boost the cleavage rate and development of embryos [33]. For example, the addition of LPA into the medium can increase the cleavage rate and promote the development of mouse [34], porcine [35,36] and bovine [37,38] embryos. Consistent with these findings, our study observed that the application of LPA to the culture medium correlated with elevated cleavage and blastocyst development rates (Table 1). Our results found an improvement in the blastocyst rate following supplementation with DKK1 in the IVC medium. Similarly, previous research had reported a tendency for increased cleavage and blastocyst rates in bovine embryos treated with DKK1 [14,39]. Moreover, when LPA and DKK1 were used in combination, there was a notable enhancement in the developmental competence of bovine blastocysts. In essence, LPA and DKK1 exert a positive influence on early embryonic development, extending to the blastocyst stage, and thereby improving in vitro production (IVP) systems.

Yes-associated protein (YAP) is a signaling and transcriptional effector that is repressed by the Hippo pathway and induces pluripotency in embryonic stem cells [40]. YAP also plays a key role in regulating the differentiation of the TE and ICM lineages’ specification during porcine [41] and mouse [42] embryonic development. Studies have further elucidated YAP function, demonstrating that LPA activates YAP/TAZ and enhances YAP/TAZ signaling to promote cell proliferation and stemness maintenance [43,44]. Aligning with these findings, our experiments revealed a significant increase in YAP fluorescence intensity in embryos following LPA treatment, likely due to LPA-mediated inhibition of the Hippo pathway kinase LATS1/2, as previously reported [40,45]. Collectively, these data support the model that LPA acts through YAP/Hippo signaling to modulate cell fate decisions and embryonic patterning.

The transcription factor CDX2 plays a pivotal role in the specification of TE and the subsequent formation of blastocysts [46]. In our experiments, treatment of preimplantation embryos with LPA, DKK1 or both LPA and DKK1 resulted in an increase in total cell numbers, TE cell numbers and the ratio of TE cells to ICM cells compared to untreated control embryos. These findings aligned with previous work by Liu et al. which demonstrated that supplementation with LPA enhanced blastocyst differentiation, as evidenced by accelerated blastocyst outgrowth rates in vitro [47]. In the presence of LPA, embryos showed higher levels of CDX2 expression, which is regulated by the Hippo pathway [48]. In mouse [49] and bovine [50] embryos, nuclear CDX2 accumulation is dependent on YAP. Likewise, Zhang et al. [35] demonstrated that LPA supplementation in the culture medium significantly enhanced the total cell count and the number of TE cells in porcine blastocysts. 

Furthermore, inhibition of the Wnt signaling pathway contributes to an increase in TE cell numbers and the total cell numbers of the blastocyst [51]. The likelihood is that DKK1 increases the expression of transcription factors that promote cell differentiation in the TE lineage [52]. Treatment of embryos with DKK1 from day 5 of development encourages the proliferation of the TE cell lineage in porcine [53], bovine [14,52,54,55] and other mammalian blastocysts. Aligning with these prior observations, our current results revealed that both TE cell lineage allocation and total cell numbers are increased following combined LPA and DKK1 treatment of bovine embryos (Table 2). Similarly, the expansion of the CDX2-positive TE cells in response to LPA and DKK1 (Figure 1) provides additional evidence supporting the interaction between Hippo and Wnt signaling during TE specification in the developing mammalian embryo. Further work is required to determine how LPA and DKK1 regulate the expression of genes involved in blastocyst differentiation. 

Apoptosis is a process of programmed cell death that is involved in the homeostasis maintenance of many biological systems [56]. Recent studies have shown that LPA treatment increased the *BCL2* and *BCL2L1* expression and decreased the *BAX* and *BAK* expression of embryos [33,37]. Like earlier studies demonstrating that LPA supplementation decreases apoptotic cell numbers in porcine blastocysts [35,37], our current experiments revealed a significant decrease in the incidence of apoptosis in the LPA group and the LPA + DKK1 group, compared to the control and DKK1 groups. Collectively, these findings provide evidence that LPA and DKK1 act cooperatively to orchestrate gene regulatory networks governing cell fate decisions in the preimplantation embryo.

In the present investigation, scRNA-seq was utilized to perform a comprehensive transcriptomic analysis at the single-embryo level for both control group and LD group embryos. Several genes related to embryo differentiation (*WNT3A*, *AMOT*) and endometrium (*MX2*, *c15H11orf34*, *MUC1*) were found to be upregulated in the LD group compared to the control group. *WNT3A* is a classical canonical WNT ligand that plays an important role in embryonic developmental processes [54] and bovine TE development and differentiation [7]. It has been demonstrated that *WNT3A* regulates *CDX2* through the regulation of the WNT-YAP/TAZ signaling pathway [57]. *AMOT* activates the Hippo signaling pathway during mouse preimplantation embryo development [58] and plays an important role in TE formation and function in bovine blastocyst development in vitro [50]. *MX2* is an optimal gene for distinguishing pregnancy, which may trigger the migration of maternal neutrophils to the developing embryo [59]. *MX2* gene expression is upregulated in early pregnancy and has an important role in regulating endometrial production, uterine recontouring and resistance to luteolysis activity [60]. *c15H11orf34* is highly upregulated in endometrium of maternally pregnant bovines [61]. *MUC1* is a cell surface glycoprotein which is expressed predominantly in the luminal epithelium of the mammalian endometrium and plays an important role in embryonic implantation and placentation [62,63]. The transcription of endometrial-related genes plays a critical role in the further development of the embryo and ultimately in the success of pregnancy [64]. 

The enrichment analysis of DEGs highlighted a significant overrepresentation of several signaling pathways, including Wnt, Notch, TGF-beta, mTOR, MAPK, ECM and Hippo. Notch has been detected in human, mouse and bovine preimplantation embryos [1,65]. The Notch pathway plays a key role in the first lineage specification and regulates TE-specific expression in mouse blastocysts [66,67], which explained the increased *CDX2* expression in the LPA and DKK1 group. The TGF-beta signaling pathway is of notable importance in mediating cellular proliferation and differentiation, both of which are essential for uterine growth and the progression of fetal development [68]. Concurrently, the mTOR pathway promotes cell proliferation during the early stages of embryonic development [69]. Embryos deficient in mTOR signaling in mouse exhibit developmental failure around implantation, highlighting its essential role in mammalian embryogenesis [70,71]. It was found that pre-implantation inhibition of mTOR significantly reduced the ratio of developing embryos to blastocysts and impaired blastocyst quality as well as the proliferation and differentiation of TE cells [72]. Moreover, the MAPK signaling pathway is involved in cell proliferation, differentiation and apoptosis [73]. Additionally, MAPK plays an important role throughout preimplantation embryonic development [74] and is involved in embryonic and yolk sac angiogenesis during fetal placenta development [75]. Furthermore, the ECM signaling pathway is recognized as a key contributor to the development of the bovine placenta as well [76]. Alterations in the ECM interface that interact with stem cells are known to influence lineage commitment directly [77]. Collectively, these significantly enriched gene signaling pathways have been identified as integral to placental and embryo development. Since our work primarily focused on the early stages of embryonic development, more investigation is needed to determine the combined impact of the mTOR, MAPK and ECM signaling pathways during the preimplantation and placental development stages in bovine.

## 4. Materials and Methods

Animals were treated according to the requirements of the Institutional Animal Care and Use Committee of the Chinese Academy of Agricultural Sciences.

### 4.1. In Vitro Maturation of Oocytes

Bovine ovaries were collected from the slaughterhouse and transported to the laboratory within 2 h at 30–35 °C. Cumulus-oocyte complexes (COCs) were collected from 2–8 mm follicles by aspiration with an 18-gauge needle. COCs were washed and those with at least 3 layers of cumulus cells were chosen for the experiment. The IVM medium contained TCM199 (Gibco, Life Technology, Carlsbad, CA, USA) supplemented with 10% fetal bovine serum (FBS, Hyclone; Gibco, Life Technology, Carlsbad, CA, USA), 1 µg/mL estradiol, 40 ng/mL insulin-like growth factor (IGF, Sigma-Aldrich, St. Louis, MO, USA), 10 µg/mL follicle-stimulating hormone (FSH, Sigma-Aldrich, St. Louis, MO, USA), 50 ng/mL epidermal growth factor (EGF, Sigma-Aldrich, St. Louis, MO, USA) and 10 µg/mL lutrinizing hormone (LH, Sigma-Aldrich, St. Louis, MO, USA). 

### 4.2. In Vitro Fertilization (IVF) of Oocytes

The IVF procedure was that mature oocytes were washed twice or three times in Brackett and Oliphant (BO) [78] fertilization medium which contained 4 mg/mL fatty acid-free BSA (Sigma-Aldrich, St. Louis, MO, USA) and 10 μg/mL heparin (Sigma-Aldrich, St. Louis, MO, USA). Oocytes were transferred into a 90 μL drop of BO fertilization medium at a density of 20–25 COCs per group. Frozen semen was thawed in a water bath at 37 °C for 30 s, rinsed twice in 7 mL BO medium containing 4 mg/mL fatty acid-free BSA and 10 mmol/L caffeine (Sigma-Aldrich, St. Louis, MO, USA) by centrifugation at 1500 rpm for 5 min, and finally resuspended at a concentration of 1 × 10^6^ spermatozoa per mL. For IVF, a 10 μL aliquot of the sperm suspension was added to each fertilization drop and the mixture was incubated for 16–18 h at 38.5 °C in humidified air containing 5% CO_2_. 

### 4.3. IVC

After fertilization, cumulus cells and the sperm were stripped from the presumptive zygotes. The zygotes were washed twice and randomly cultured in different groups of 25 in droplets of 100 µL CR1aa medium supplemented with 6 mg/mL BSA at 38.5 °C under 5% CO_2_. The experiment was divided into four groups: (i) control group: culture in CR1aa medium; (ii) LPA group: CR1aa medium supplemented with 10^−5^ M LPA (Sigma-Aldrich, St. Louis, MO, USA) for 48 h at 38.5 °C under 5% CO_2_; (iii) DKK1 group: on day 5 after initiation of fertilization, CR1aa medium supplemented with 10% FBS and 100 ng/mL DKK1 (Sigma-Aldrich, St. Louis, MO, USA) for 48 h at 38.5 °C under 5% CO_2_; (iiii) LPA + DKK1 group: CR1aa medium supplemented with 10^−5^ M LPA and 100 ng/mL DKK1. Embryos were cultured at 38.5 °C under 5% CO_2_ until day 7 after initiation of fertilization. The cleavage rate was evaluated at day 2 and blastocyst rate was evaluated at day 7. 

### 4.4. Immunofluorescence Staining of Embryo

Immunofluorescence was performed according to Yu et al. [79] with some modifications. The embryos were fixed in 4% paraformaldehyde for 1 h at 4 °C. Subsequently, the embryos were permeated with 0.5% Triton X-100 (Sigma-Aldrich, St. Louis, MO, USA) at room temperature for 40 min. Then, the embryos were blocked with PBS containing 1% BSA at 4 °C overnight. The embryos were moved into the primary antibodies YAP (1:500, abcam, Cambridge, UK) and CDX2 (1:500, abcam, Cambridge, UK) diluted with 1% BSA at 4 °C overnight, and then washed three times with 0.5% Triton X-100 (Sigma-Aldrich, St. Louis, MO, USA) for 10 min each. Embryos were incubated with the secondary antibodies Alexa Fluor 594 anti-mouse (1:1000, abcam, Cambridge, UK) and Alexa Fluor 488 anti-rabbit IgG (1:1000, Cell Signaling Technology, Danvers, MA, USA) at room temperature for 1 h in the dark. Finally, nuclei were stained with 5 mg/mL DAPI (Beyotime, Shanghai, China) for 10 min in the dark at room temperature. The fluorescent images were obtained by a confocal microscope (Leica, Wetzlar, Germany) and analyzed using Image J software version 1.8.0 (NIH, Bethesda, MD, USA). The total cell number of individual embryos was measured by DAPI (Beyotime, Shanghai, China) staining, and the number of putative TE cells was determined by CDX2 positive staining.

### 4.5. Detection of DNA Fragmentation by TUNEL 

Apoptosis was analyzed using a TUNEL (terminal deoxynucleotidyl transferase biotin-dUTP nick end labeling) assay kit (Solarbio, Beijing, China) following the manufacturer’s instructions. In brief, blastocysts were washed three times with PBS containing 0.1% PVA (PBS-PVA), fixed with 4% paraformaldehyde and permeabilized by incubation in 0.1% Triton X-100 (Sigma-Aldrich, St. Louis, MO, USA) for 30 min at room temperature. Next, the embryos were washed three times with PBS-PVA and incubated with fluorescein-conjugated dUTP and the terminal deoxynucleotidyl transferase enzyme in the dark for 1 h at 37 °C. After incubation with 1 mg/mL Hoechst 33,342 for 5 min at 37 °C to label the nuclei, the samples were fixed on slides and examined by confocal microscope (Leica, Wetzlar, Germany). The total cell number of blastocysts (DAPI) and the number of apoptotic cells of blastocysts (TUNEL) were counted using ImageJ software version 1.8.0 (NIH, Bethesda, MD, USA). The apoptosis rate of blastocysts = the number of apoptotic cells of blastocysts/total cell number of blastocysts.

### 4.6. Sample Preparation, Library Construction and RNA Sequencing 

The embryo zone pellucida was removed by 5 mg/mL pronase for 1–2 min. Each embryo was pooled and dissolved in lysis buffer. The protocol for scRNA-seq library preparation and sequencing was performed according to the method described by Gao et al. [80] with some modifications. A single cell was lysed to release RNAs, which were then reverse transcribed into first-strand cDNAs using six-base random primer random hexamers. The second strand cDNA was synthesized from dNTPs in the DNA polymerase I system, and then the double-stranded cDNA was purified by AMPure P beads. Finally, purified single-cell cDNAs were prepared for sequencing libraries according to Illumina’s TruSeq DNA sample preparation protocols. For the qualified cDNA libraries, sequencing was performed by the Illumina platform, and the PE150 sequencing strategy was run to obtain 150-bp double-ended sequencing reads.

### 4.7. Functional Enrichment Analysis

In the present study, gene ontology (GO) enrichment analysis was performed based on three terms including biological processes, cellular components and molecular functions (http://www.geneontology.org/), accessed on 20 October 2024). Kyoto Encyclopedia of Genes and Genomes (KEGG) enrichment analysis distinguished significantly enriched biological pathways. Significance was set at a *p*-value of <0.05. 

### 4.8. Statistical Analysis

All experiments were repeated at least three times, and all data were presented as mean ± standard error. All analyses were performed using SAS software version 9.2.0 (SAS Institute, Carrey, NC, USA) to compare the groups. Differences were regarded to be significant when *p* < 0.05. 

## 5. Conclusions

In the context of our investigation, the addition of LPA and DKK1 was found to have remarkable results in improving early embryo quality. This improvement was observed to be intricately associated with its regulatory influence on the TE lineage, modulation of apoptosis and its interactive engagement with critical signaling pathways, including the Hippo, Wnt and MAPK pathways, during the in vitro development of bovine embryos.

## Figures and Tables

**Table 1:** Actions of LPA and DKK1 on the developmental ability of bovine embryos.

Groups	NO. of COCs	NO. of Embryos Cleaved	Blastocyst Rate
Control	133	99 (74.18 ± 3.44) ^c^	30 (30.02 ± 3.30) ^c^
LPA	138	115 (83.57 ± 3.60) ^b^	46 (40.04 ± 2.54) ^b^
DKK1	145	119 (81.97 ± 3.38) ^b^	46 (38.91 ± 3.10) ^b^
LPA + DKK1	127	114 (89.67 ± 4.53) ^a^	54 (47.11 ± 4.23) ^a^

^a^, ^b^, ^c^ Values with different superscripts indicate significant difference between groups (*p* < 0.05).

**Table 2:** Summary of raw and clean reads of mRNA.

Index	Sample					
	Con1	Con2	Con3	LD1	LD2	LD3
Raw Reads/bp	63,756,616	59,220,724	45,639,012	55,652,282	53,065,386	41,746,418
Clean Reads/bp	62,536,622	58,162,374	44,891,384	54,470,954	52,075,814	40,869,626
Clean Reads Rate (%)	98.09	98.21	98.36	97.88	98.14	97.90
Clean Q30 (%)	96.00	95.97	95.89	96.41	96.11	96.13
Mapped Reads/bp	60,980,959	56,889,681	43,953,245	53,453,414	50,735,875	39,926,192
Mapping Rate (%)	97.51	97.81	97.91	98.13	97.43	97.69

**Table 3:** Statistics of different expression of mRNA of LD and Control groups.

Gene	Sample						Up/Down
	LD1	LD2	LD3	Con1	Con2	Con3	
*GPX3*	5	47	38	6	3	0	up
*SORBS3*	74	179	267	10	29	40	up
*MX2*	26	286	74	0	25	0	up
*FAM46A*	748	710	172	57	198	107	up
*FOS*	3482	9111	5408	1770	777	2426	up
*ASTL*	17	36	127	5	0	12	up
*ID1*	51	220	98	13	6	9	up
*MAP1LC3A*	363	248	214	138	130	133	up
*C15H11orf34*	293	5806	473	11	69	8	up
*SOX6*	79	20	17	11	14	79	up
*OAS1Y*	25	152	0	0	0	0	up
*RICTOR*	1089	1323	1061	748	924	432	up
*TUNAR*	14	53	0	0	0	0	up
*TGFBR2*	99	308	420	192	16	56	up
*RGS4*	6960	1438	0	34	11	9	up
*FAS*	480	1454	1385	357	190	0	up
*MUC1*	992	1502	556	50	294	34	up
*OVOL1*	475	553	292	86	288	48	up
*BEX2*	17	187	7	7	3	3	up
*AMOT*	2114	2437	727	276	1038	216	up
*IFNGR2*	8	197	41	0	1	0	up
*ABCB4*	150	450	27	2	9	0	up
*IL1RAP*	18	140	106	33	33	17	up
*KRT7*	2200	5046	7411	3081	2034	1022	up
*MGST1*	434	1348	58	31	248	31	up
*IGFBP7*	22	108	118	8	2	0	up
*WNT3A*	4	33	12	0	0	0	up
*COL5A3*	17	111	44	7	12	1	up
*HMGB2*	3389	2589	2258	6630	4773	5665	down
*OTX1*	37	22	0	282	2	1442	down
*RSPO3*	268	230	77	523	380	680	down
*RASSF2*	50	65	52	1377	15	799	down
*LRP4*	63	38	12	61	94	291	down
*SLC25A27*	105	54	47	464	190	153	down
*GCM1*	32	2	0	117	320	90	down
*BAD*	331	513	326	1242	398	1843	down
*FAM53B*	23	4	1	53	41	64	down
*EED*	1068	704	811	2493	1260	3958	down
*WNT2*	0	0	0	0	4	64	down
*NANOG*	185	381	68	50	2645	14,549	down

## Data Availability

All the data supporting the conclusions in this article have been presented in the manuscript.

## Supplementary Materials

The following supporting information can be downloaded at: https://www.mdpi.com/article/10.3390/ijms25073912/s1.
